# Psychosocial Dimensions of Recycling in Small Island: Psycho-Environmental Diagnostic and Evidence-Based Intervention in Martinique

**DOI:** 10.3389/fpsyg.2022.908631

**Published:** 2022-07-08

**Authors:** Valérie Fointiat, Audrey Pelt

**Affiliations:** ^1^Aix-Marseille University, LPS, Aix-en-Provence, France; ^2^University of Lorraine, PErSEUs, Metz, France

**Keywords:** recycling, attitude, norm, identity, behavioral control, behavioral change, sense of community, temporal distance

## Abstract

Recycling is socially responsible behavior. Moreover, it is also a complex behavior. It benefits society in the long term but involves a personal cost and does not benefit the individual directly. In the specific context of small island, we conducted a two-step research action to promote recycling among households located in the north of Martinique, a west indies French island. Based on the psychosocial engineering model, the first step was to identify the determinants of recycling in this specific island context. In a psycho-environmental diagnostic, we included variables already identified to predict waste sorting, such as the TPB variables, as well as other context-specific variables, such as group identification, environmental identity, place identity, sense of community, perceived efficacy of one’s action, as predictors of the intention to recycle. Based on psychological distance and its temporal dimension, we also distinguished between the intention to recycle today (proximal intention) and the intention to recycle in 1 month (distal intention). The results show that the predictors of recycling differ according to the temporal distance. The proximal intention was predicted by personal variables whereas distal intention was predicted by social variables. The second step was to design and to implement an evidence-based intervention to increase the quality of recycling. At Time 1, the garbage cans of 193 households were collected and characterized. At Time 2, the households were visited at home by an officer, who used one of the four communication scripts built on the basis of the results of the psycho-environmental diagnostic. At Time 3, the garbage cans were collected and characterized again. We observe an improvement in sorting in the condition where the TBP dimensions are activated: attitude, social norm, and controllability. Our results highlight the importance of taking into account the temporality of change, especially when the behavior to be changed is complex. They also show an interest in relying on a psycho-environmental diagnosis, taking into account the context of insertion of the target behavior.

## Introduction

Solid waste has a strong negative impact on the environment: it damages soils and beaches and contributes to marine pollution (United Nations Commission on Sustainable Development, 1999). The management of solid waste is a major challenge for island territories. Solid waste management policies come up against heavy constraints, particularly where landfill is concerned as it involves limited spatial availability (Deschenes and Chertow, 2004). This constraint is all the heavier for territories with a small surface area and a large population density, as is the case for the French West Indies. Solid waste management policies can set up legal and technical solutions, for example, by widening the perimeter of the collection zones or by increasing the recyclable waste drop-off points. These solutions are, however, not sufficient in themselves if they are not supported by the citizens (Dai et al., 2015). The citizens themselves carry out the primary sorting of waste by separating recyclable waste from other types of waste, preparing it if necessary (washing) and placing it in a suitable garbage can or container. Ensuring citizens’ good waste-recycling practices improves sorting efficiency and reduces the costs of the whole recycling process (Miranda and Blanco, 2010). The awareness and communication campaigns aimed at promoting domestic recycling are therefore essential to support people in adopting good waste-recycling practices.

### Context of the Intervention–Recycling in Martinique

Martinique is a small volcanic island with a surface area of 1,228 square kilometers and a population of 397,357 (estimated at the beginning of 2018). Like the other French Caribbean islands of Guadeloupe and Saint-Martin (shared with the Netherlands), Martinique is 7,000 km from mainland France. Their remoteness and isolation are obstacles when setting up recyclable waste externalization policies (Eckelman et al., 2014). Moreover, under the influence of demographic expansion, urbanization, the increase in consumption, and the intensive production of plastic waste, waste volumes continue to grow. To illustrate this, the Martinicans produce over 3,100 tons of recyclable waste per year (∼7.9 kg/year/inhabitant). A study carried out in 2013 by ADEME (Agence de l’Environnement et de la Maîtrise de l’Énergie) highlighted the fact that recycling instructions do not appear to be well-understood and are not properly applied (separating of food waste, paper, and cardboard). During door-to-door collections, 18% of recyclable waste cannot actually be collected by the competent public authorities due to a too large number of sorting errors. In this context, the Communauté d’Agglomération du Pays Nord Martinique (CAP Nord) wished to implement a research-action approach to improve the quality of household selective recycling. This territorial community covers 18 communes and around one-third of the island’s surface area.

### Promotion of Recycling

To promote household recycling, two research lines emerged. The aim of the first was to identify the determinants of waste-recycling behavior. Thus, over the last 30 years, researchers have evaluated the socio-demographic, psychological, and contextual determinants of recycling, taking into account age, gender, attitudes, social norms, knowledge, affects, motivation, self-identity, values, housing situation, or local circumstances [see for a review: Hornik et al. (1995), Schultz et al. (1995), Timlett and Williams (2011), Miafodzyeva and Brandt (2013), Geiger et al. (2019), Rousta et al. (2020)]. Concurrently, the second research line aimed at accompanying change. The proposed interventions rely on strategies such as prompts and information, educational campaigns, feedback, commitments, incentives, environmental alteration, and social modeling (Varotto and Spagnolli, 2017; Seacat and Boileau, 2018; Li et al., 2019).

Nevertheless, the meta-analysis of Varotto and Spagnolli (2017) shows a tenuous link between the two research orientations. The majority of the studies reviewed which describe the determinants of waste recycling give little or no indication of efficient methods for changing behaviors. Conversely, the interventional research used to promote recycling rarely refers to the theory and specifies the underlying determinants. Varotto and Spagnolli (2017) highlight the fact that the design of interventions targeting the adoption of a behavior must be preceded by a diagnostic study to enable the identification of the determinants.

Following this recommendation, we propose to conduct this diagnostic using the variables detected previously in the literature. This research is thus based on the theory of planned behavior (TPB) which makes it possible to include the dimensions linked to identity and the sense of community which are specific to the small island context.

### Understanding the Islander’s Recycling Determinants

#### Theory of Planned Behavior and Recycling Behavior

TPB (Ajzen, 1991) is a well-known model used to explain and predict recycling behavior [for a meta-analysis, see Geiger et al. (2019)]. According to TPB, behavior is directly dependent on behavioral intention. In turn, behavioral intention is itself determined by three core constructs, namely attitude, subjective norm, and perceived behavioral control.

Attitude is based on behavioral beliefs, which are a person’s beliefs about the likely consequences of performing the behavior (Ajzen, 1991, 2005). Scientific literature examines the difference between general attitude toward environment and specific attitudes toward recycling (i.e., the way in which a person evaluates recycling favorably) (Geiger et al., 2019). For our part, we measure specific attitude toward recycling because this construct is a better predictor than general attitude. Specific attitude toward recycling is often overlooked in the design of interventions (Varotto and Spagnolli, 2017).

Subjective norm refers to the normative dimension and what the person thinks it is right to do. Norms are strongly linked to recycling (Geiger et al., 2019). The social influence and the impact of norms are concepts that are often mobilized in recycling-aimed interventions (Dupré et al., 2014).

Perceived behavioral control influences behavioral intention and also has a direct influence on behavior. Behavioral control is made up of two distinct elements (Ajzen, 2002): self-efficacy and controllability. Self-efficacy refers to the perceived ease of performing the behavior. Where recycling is concerned, the perceived ease may, for example, depend not only on the identification of products that are–or are not–recyclable but also on the presence of recycling cans in people’s homes. Controllability is defined as the degree to which a person believes that the behavior is under his/her control alone.

Where behavioral intention is concerned, if research has multiplied to understand and promote behavioral changes, we have little knowledge of the manner in which the intention of adopting a target behavior changes according to the temporality considered (Lutchyn and Yser, 2011). In addition to this, people’s intentions change with time. We have therefore taken into consideration the intention to recycling now and the intention to recycle in a month’s time.

#### Identities: Environmental Identity, Place Identity, and Group Identification

Studies indicate that the self-identity of small-island residents is likely to be an important predictor of their behavior (Nunkoo et al., 2010). The question of the weight of the identity variable in pro-environmental behaviors can be analyzed at different levels: environmental identity, place identity, and social identification.

Environmental identity reflects the extent to which a person sees him/herself as an environmentally friendly person in general (Van der Werff et al., 2013a,b; Geiger et al., 2019). In other words, it can be defined as the extent to which one sees oneself as a type of person whose actions are environmentally friendly (Devine-Wright and Clayton, 2010; Van der Werff et al., 2013a). People for whom environmentalism is a central part of who they are, are more likely to engage in pro-environmentalist actions (Manetti et al., 2004; Nigbur et al., 2010; Gatersleben et al., 2014).

Place identity is defined as a symbolic dimension of place attachment. Place identity is constructed during experiences, emotions, and constructed history. Place becomes an integral part of self-concept. Place identity and place attachment have a strong relationship with pro-environmentalist intention (Hernández et al., 2020).

Social identity (Tajfel, 1979) refers to the group as a perceived entity. As an entity, the group has its own characteristics, functions through norms, and is in relation with outgroups. In contrast, social identification refers to the individual member’s relationship with that entity. Therefore it is more individually determined. Postmes et al. (2013, p. 599) provide a Tajfelian definition of social identification, “as being the positive emotional valuation of the relationship between self and in group.” Postmes et al. (2013) proposed to measure social identification by using a single item measurement, which captures 70% of self-investment and 49% of self-definition.

#### Relationship With Others: Sense of Community in an Island Perspective

Recycling is a socially responsible behavior that is an action taken by individuals to enhance societal well-being (“do good”) or to avoid harmful consequences for the collective (“do not harm”) (Crilly et al., 2008, p. 176; Cojuharenco et al., 2016). As many other socially responsible behaviors, recycling illustrates the individual contributions to the collective good. If recycling is desirable from a collective point of view, it is often costly or inconvenient for the individual. A crucial factor in people’s decisions to recycle is the extent to which they believe that their actions make a difference (Ellen et al., 1991).

Some studies on the relationship of islanders and particularly small-island inhabitants have shown that the main point for islanders is the quality of a self-other relationship (Podgorelec et al., 2015). The authors have shown that small-island inhabitants were particularly concerned with their image and worry about what other people think of them.

Sense of community is defined as a feeling of attachment and concern for one’s community (i.e., self-connectedness), associated with the perception that their action benefits their own community (i.e., perceived effectiveness of one’s action, PEOA). To our knowledge, sense of community was not included in any of the field interventions to promote recycling whereas McCarty and Shrum (2001) demonstrated a link between the sense of connectedness and self-reported recycling behaviors. PEOA is identified as a crucial dimension of socially responsible behavior. Cojuharenco et al. (2016) consider that this dimension is positively correlated to the sense of connectedness to others: the more people feel connected to others, the more they believe that their actions have an impact on the collective good. Self-connectedness and perceived effectiveness of one’s action are variables which can be important in the island context.

In sum, we develop a comprehensive approach with two objectives: (1) to assess the links between the behavioral determinants taking into account at the same time the island context which is known to strengthen identities (Geiger et al., 2019) and the feeling of connectedness to others (McCarty and Shrum, 2001) and (2) propose concrete courses of action whose effectiveness will be tested and validated as part of a field intervention using efficient waste measurement. The strong point of this intervention is using an effective behavioral measurement (quality of recycled waste). We have chosen not to use self-reported measurements which, subjected to a strong social desirability, can lead to a gap between reported behaviors and effective behaviors (Gamberini et al., 2014).

## Psycho-Environmental Diagnostic

### Participants and Procedure

An online survey was conducted of residents of Cap Nord Martinique^1^ during May and July 2019. Of the 372 responses received, 213 were completed. The mean age of participants was 47.02 years (SD = 12.992). There were 143 females, 69 males, and one who did not indicate the gender.

### Measures

The survey comprises several sections (Table 1). In a first section, items measured knowledge of recycling. Specific knowledge about recycling is a good recycling predictor (Dupré et al., 2014; Geiger et al., 2019). A second section evaluated the key factors of the TPB model (i.e., attitude, subjective norm, and self-efficacy and controllability). The TPB components were constructed using guidelines provided by Ajzen (2006). In a third section, additional variables were rated: environmental identity, place identity, group identification, self-connectedness, and perceived effectiveness of one’s action. A last section included socio-demographical items.

**TABLE 1:** Items measured in the psycho-environmental diagnostic.

Dimensions	Items
Specific attitude to recycling	I think that sorting my waste is: bad/good, useless/useful, clean/dirty, disgusting/not disgusting, odorous/odorless, time consuming/quick to do, irresponsible/responsible, and bulky/non-bulky
Subjective norm	Most of the people who are important to me think that I should recycle, Most of the people who are important to me think that I should commit to recycling, Most of the people who are important to me approve of the fact that I recycle
Self-efficacy	It’s easy for me to know which waste to recycle, It’s easy for me to store the waste to be recycled in my home, It’s easy for me to take out the garbage can.
Controllability	I feel capable of recycling, I am confident that I can recycle.
Behavioral intention	I intend to recycle during the coming month, I will recycle starting today.
Environmental identity	I see myself as someone who respects the environment, I see myself as someone who is very concerned by environmental issues, It would embarrass me to be seen as someone with an environmentally friendly lifestyle, I would not like my family to think of me as someone who is concerned by environmental issues
Place identity	My quarter is very special for me, Visiting my quarter says a lot about who I am, I identify strongly with my quarter, I am very attached to my quarter
Self-connectedness	Caring deeply about another person such my neighbor is important for me, Caring deeply about another person such a close friend is important for me, When I become involved in a group project, I do my best to ensure its success, It is important to me that I uphold my commitments to significant people in my life
Perceived effectiveness of one’s action	It is worthless for the individual consumer to do anything about pollution, Since one person cannot have any effect upon pollution, it does not make any difference what I do, Every time people sort, it has a positive effect on society.
Group identification	I identify with the Martinicans.
Income level	The average monthly income per household in Martinique is around 1,400 € (nets). If we consider the incomes of all the members of your household, is your income: much lower to much higher than the average monthly income?

#### Section 1: Recycling Knowledge

Adapted from Dupré et al. (2014), the respondents had to choose from four cans (household waste, recyclable waste, glass, and bio-waste), the can in which they throw away each of the twenty waste items presented (e.g., pizza carton and plastic bottle). A knowledge score out of twenty is calculated by adding the right answers (coded 1).

#### Section 2: Theory of Planned Behavior Variables

##### Specific Attitude Toward Recycling

Attitude toward household waste recycling was measured with the statement “I think recycling my waste is …” followed by eight bipolar adjectives (good/bad) separated by a seven-point scale. The data were aggregated in an attitude score (α_Cronbach_ = 0.88).

##### Subjective Norm

Three items measured the subjective norm (e.g., “Most of the people who are important to me think that I should recycle”). The respondents indicated their degree of agreement on a 7-point Likert scale. A factorial analysis (KMO = 0.625, *p* = 0.000, Bartlett < 0.000) confirmed its one-dimensional nature. The three items account for 76.62% of explained variance. Consequently, an overall score was calculated by aggregating the data (α_Cronbach_ = 0.85).

##### Self-Efficacy

Self-efficacy is measured by three items (e.g., “it is easy for me to store the waste to be recycled in my home”). We thus constructed a self-efficacy score (α_Cronbach_ = 0.81).

##### Controllability

Controllability is measured by two items (e.g., “I feel capable of recycling”) which correlate positively and significantly (*r*_Pearson_ = 0.698, p = 0.000). We thus aggregated the data from the two items to construct a score.

##### Behavioral Intention

As recycling is a complex behavior, which may necessitate some time to set up, we have chosen to measure on a seven-point scale the intention to recycle in the short term (“starting today”) and in the medium term (“during the coming month”).

#### Section 3: Additional Variables

##### Environmental Identity

Environmental identity was measured using four items adapted from Sparks and Shepherd (1992), Cook et al. (2002), and Whitmarsh and O’Neill (2010). The factorial analysis confirms a two-dimensional structure. The first dimension covers two items and explains 43.17% of the total variance. We therefore built a self-oriented environmental identity index (e.g., “I see myself as someone who respects the environment,” “I see myself as someone who is very concerned by environmental issues”). These items are significantly correlated (*r*_Pearson_ = 0.72, *p* = 0.000). The second dimension explains 36.79% of the total of variance and refers to other-oriented environmental identity (e.g., “It would embarrass me to be seen as someone with an environmentally friendly lifestyle,” “I would not like my family to think of me as someone who is concerned by environmental issues,” reversed score), *r*_Pearson_ = 0.477, *p* = 0.000.

##### Place Identity

Place identity was measured by four items adapted from Williams and Vaske (2003) (e.g., “I identify strongly with my quarter,” “I am very attached to my quarter”). We built an index by aggregating the data (α_Cronbach_ = 0.89).

##### Sense of Community

###### Self-Connectedness

Self-connectedness (Cojuharenco et al., 2016) was measured in four items (i.e., “caring deeply about another person such my neighbor is important for me”). We thus built an overall self-connectedness index of (α_Cronbach_ = 0.88).

###### Perceived Effectiveness of One’s Action

To measure the perceived effectiveness of having a positive impact on society and the environment, the participants completed three items adapted from Cojuharenco et al. (2016) (i.e., “Since one person cannot have any effect upon pollution, it does not make any difference what I do”). We thus aggregated the data to build an index (α_Cronbach_ = 0.62).

###### Group Identification

Group identification was measured by using SISI (Single Item Social Identity, Postmes et al., 2013). Taking Martinique’s specificity into consideration, we proposed an item: “I identify with Martinicans.” The respondents answered on a 7-point scale, from 1 not at all to 7 completely.

#### Section 4: Socio-Demographic Variables

In a last section, the respondents had to indicate their gender, their date of birth and their place of residence. Lastly, a question dealt with the average income per household. The participants indicated on a 7-point scale if they earned well below or well above the Martinican average monthly income estimated at 1,400 Euros/month.

## Results

Two series of multiple regressions were conducted (stepwise method) to predict behavioral intention to recycle starting today (VD1) and to predict behavioral intention to recycle in 1 month (VD2). Twelve predictors were entered into the model to predict each of these two variables: recycling knowledge, income per household, specific recycling attitude, subjective norm, self-efficacy, perceived behavioral control, personal environmental identity, social environmental identity, perceived effectiveness of one’s action, place identity, self-connectedness, and group identification as a Martinican.

### Determinants of the Intention to Recycle Waste Starting Today

Out of the 213 complete questionnaires,^2^ we excluded four outliers at ±3 SD, that is, a final sample of 209. We also checked for a possible colinearity, both by scanning the correlation matrix and completing this analysis by the VIF (variance inflation factor) and its reciprocal the Tolerance statistic (1/VIF). The matrix scan indicated the strongest correlation at *r* = 0.61, which is far from the minimum level set at 0.80 or 0.90 (Field, 2009). The mean VIF is around 1, and Tolerance at 0.72. There is thus probably no collinearity (Ménard, 1995).

The determinants (Table 2) that weigh the most on this intention of immediate action refer to self-oriented environmental identity (Bêta = 0.27, *t* = 4.318, *p* < 0.001, IC = [0.190; 0.510]) and to the belief that has their own capacity to recycle (self-efficacy *B* = 0.24, *t* = 3.499, *p* < 0.001, IC = [0.098; 0.350] and controllability (Bêta = 0.14, *t* = 2.129, *p* < 0.05, IC = [0.013; 0.345]) and the belief that together we can act effectively (perceived efficacy of one’s action, Bêta = 0.12, *t* = 2.090, *p* < 0.05, IC = [0.009; 0.303]). Recycling knowledge is also a predictor of the intention to recycle starting today, even if its weight is less in comparison with the other factors (Bêta = 0.17, *t* = 2.981, *p* < 0.003, IC = [0.026; 0.128]).

**TABLE 2:** Multiple regression: behavioral intention to recycle starting today.

		B	SE B	β
Step 1	Constant	1.81	0.47	
	Self-oriented environmental identity	0.71	0.07	0.55***
Step 2	Constant	1.36	0.44	
	Self-oriented environmental identity	0.50	0.08	0.39***
	Self-efficacy	0.34	0.06	0.36***
Step 3	Constant	0.74	0.47	
	Self-oriented environmental identity	0.44	0.08	0.34***
	Self-efficacy	0.30	0.06	0.32***
	Knowledge	0.08	0.03	0.19**
Step 4	Constant	0.26	0.50	
	Self-oriented environmental identity	0.34	0.08	0.31***
	Self-efficacy	0.23	0.06	0.24**
	Knowledge	0.08	0.03	0.18**
	Controllability	0.20	0.08	0.16*
Step 5	Constant	0.231	0.55	
	Self-oriented environmental identity	0.35	0.08	0.27***
	Self-efficacy	0.22	0.06	0.24**
	Knowledge	0.08	0.03	0.17**
	Controllability	0.18	0.08	0.14*
	Perceived efficacy of one’ action	0.16	0.07	0.12*

*R^2^ = 0.30 for step 1, R^2^ = 0.11 for step 2 (p < 0.001), R^2^ = 0.03 for step 3 (p < 0.01), R^2^ = 0.15 for step 4 (p < 0.02), R^2^ = 0.17 for step 5 (p < 0.03).*

*p < 0.05. **p < 0.01. ***p < 0.001.

### Determinants of the Intention to Recycle Waste During the Coming Month

Out of the 213 complete questionnaires, we excluded five outliers at ±3 SD, that is, a final sample of 208. We also checked for a possible collinearity, both by scanning the correlation matrix and by completing this analysis by the VIF (variance inflation factor) statistic and its reciprocal the Tolerance statistic (1/VIF). The scan of the matrix indicates a strong correlation at *r* = 0.61, which is far from the minimum level set at 0.80 or 0.90 (Field, 2009). The mean VIF is around 1, and Tolerance at 0.72. There is thus probably no collinearity (Ménard, 1995).

The intention to recycle in the coming month is predicted by six factors (Table 3). Self-efficacy is the strongest predictor (Bêta = 0.26, *t* = 4.15, *p* < 0.001, IC = [0.129; 0.364]): the more the respondents deem that recycling is easy for them, the more they consider recycling in the coming month. Another set of factors related to social relationships also predict behavioral intention: self-connectedness (Bêta = 0.16, *t* = 2.37, *p* < 0.02, IC = [0.039; 0.425]), group identification (Bêta = 0.17, *t* = 2.782, *p* < 0.01, IC = [0.042; 0.248]), and subjective norm (Bêta = 0.16, *t* = 2.55, *p* < 0.01, IC = [0.031; 0.238]). In addition to this, the specific attitude toward recyling also predicts the intention to recycle in a long term, but this factor is the one that weighs the least in the intention to recycle (Bêta = 0.12, *t* = 1.973, *p* < 0.05, IC = [0.000; 0.317]). Lastly, the household’s income predicts the intention to sort (Bêta = 0.16, *t* = 2.655, *p* < 0.01, IC = [0.039; 0.262]).

**TABLE 3:** Multiple regression—behavioral intention to recycle in the coming month.

		B	SE B	β
Step 1	Constant	4.08	0.31	
	Self-efficacy	0.40	0.06	0.43***
Step 2	Constant	1.99	0.56	
	Self-efficacy	0.32	0.06	0.34***
	Self-connectedness	0.40	0.09	0.28***
Step 3	Constant	1.43	0.59	
	Self-efficacy	0.29	0.06	0.31***
	Self-connectedness	0.40	0.09	0.28***
	Income	0.15	0.06	0.16**
Step 4	Constant	1.15	0.60	
	Self-efficacy	0.29	0.06	0.31**
	Self-connectedness	0.33	0.09	0.23**
	Income	0.16	0.06	0.17**
	Group identification	0.12	0.05	0.14*
Step 5	Constant	0.85	0.60	
	Self-efficacy	0.26	0.06	0.28***
	Self-connectedness	0.27	0.10	0.19**
	Income	0.16	0.06	0.17**
	Group identification	0.14	0.05	0.16**
	Subjective norm	0.13	0.05	0.15*
Step 6	Constant	0.26	0.67	
	Self-efficacy	0.25	0.06	0.26***
	Self-connectedness	0.23	0.10	0.16*
	Income	0.15	0.06	0.16**
	Group identification	0.14	0.05	0.17**
	Subjective norm	0.13	0.05	0.16*
	Attitude	0.16	0.08	0.12*

R^2^ = 0.18 for step 1, R^2^ = 0.07 for step 2 (p < 0.001), R^2^ = 0.10 for step 3 (p < 0.01), R^2^ = 0.12 for step 4 (p < 0.02), R^2^ = 0.14 for step 5 (p < 0.01), R^2^ = 0.15 for step 6 (p < 0.05). *p < 0.05. **p < 0.01. ***p < 0.001.

## Discussion

Our aim was to establish a psycho-environmental diagnostic using TPB model including psychosocial identities and variables specific to the island context such as sense of community, taking into consideration at the same time the temporal dimension associated with behavioral intention. Lutchyn and Yser (2011) associated TPB with construal level theory (Liberman and Trope, 2003; Trope and Liberman, 2003) to understand the influence of temporal distance. Using belief-elicitation research, Lutchyn and Yser (2011) demonstrated that the temporal perspective affects the type of salient behavioral beliefs, in such a way as people generate more feasibility beliefs (i.e., self-efficacy) by thinking of proximal behaviors, but more desirability (i.e., attitude and normative) when the behavior is distal. Construal level theory postulates that temporal distance changes the way in which people see an action. This theory also postulates that temporal distance has an influence on social distance (me vs. others), in terms of identity. A distal temporal distance is associated with abstract constructs, whereas a proximal temporal distance implies concrete construal. In a context of behavioral change, this means that when the target behavior is temporally distal, people concentrate more on the why of the action and on its desirability. Conversely, when the behavior takes place in the immediate future, people will focus on the how of the behavior, and on the feasibility. In line with this reasoning, our results show that short- and medium-term behavioral intention is based on different factors, with the exception of self-efficacy. When the respondents project themselves into a proximal future, their intention is defined by self-oriented environmental identity, self-efficacy, controllability, knowledge, and perceived efficacy of one’s action. Taken altogether, these determinants highlight the articulation of the feasibility (knowledge, self-efficacy, controllability, and perceived efficacy of one’s action) and self-orientation (self-oriented environmental identity). We know that temporal distance is intrinsically associated with social distance (Passafaro et al., 2019). A proximal temporal distance leads the person to focus on aspects of self and on their identity.

When the respondents project themselves into a distal future, their intention is predicted by specific attitude toward recycling, subjective norm, group identification, self-connectedness, and self-efficacy. These determinants refer to the why of recycling (attitude) and its desirability (subjective norm), and other-orientation (group, identification, connectedness). A distal temporal distance is associated with a distal social distance (Passafaro et al., 2019), which leads the person to focus on others. This gives a feeling of being connected with my relatives, my neighbors, my identity as a Martinican, to the weight I grant to what others think of me and my intention to sort waste. Where the weight of self-efficacy is concerned, our results show that in a distal future, people can also refer to behavioral control beliefs. People’s belief in their own sorting capacity is decisive in the adoption of recycling behavior (Geiger et al., 2019). Lastly, the weight of income on the intention to sort later. Areke (2004) shows that sorting in households is linked to income: modest households sort less than the high-income households.

In conclusion, and as part of a research action aiming at accompanying change, it appears essential to us to take into consideration the temporality of change in which the participants find themselves. Since recycling intentions are defined differently according to these temporalities, we have articulated determinants of the proximal and distal intention for each of our interventions. The effectiveness of these interventions was evaluated with the measurement of effective behavioral change.

## Psycho-Environmental Intervention: Promote Recycling Behavioral Change

We aim to compare four original interventions, each one of them embodying one or more proximal and distal behavioral determinants identified by the psycho-environmental diagnostic (Table 4).

**TABLE 4:** Behavioral determinants highlighted in each of the interventions.

	Determinants of intention to recycle starting now	Determinants of intention to recycle in the coming month
1. Information-based int.	Knowledge	—
2. Connectedness-based int.	Perceived efficacy one’s action	Self-connectedness
3. Identity-based int.	Self-efficacy Self-oriented environmental identity	Self-efficacy Group identification
4. TPB-based int.	Controllability	Attitude toward recycling Subjective norm

The information-based intervention provides information that increases the level of knowledge. The connectedness-based intervention stresses self-connectedness and perceived efficacy of one’s action. The identity-based intervention highlights both self-efficacy and identity. Identity refers to self-oriented environmental identity as an aspect of self-identity, and Martinican group identification as group identity. The TPB-based intervention underlines the quality and quantity of recycling (i.e., controllability), specific attitude, as well as social pressure to comply with behavior (subjective norm). Subjective norm was operationalized by social gaze which is known to trigger pro-social behavior (Conty et al., 2016). Social gaze results in a motivation to avoid breaching social norms (Oda et al., 2015).

### Method

#### Overview

**FIGURE 1:**
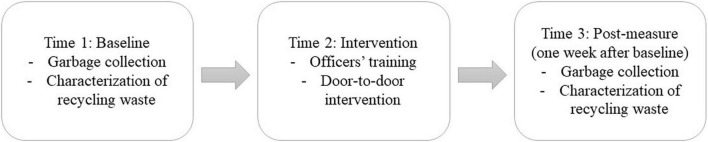
Overview of psycho-environmental intervention.

Psycho-environmental intervention took place for 2 weeks, between October 14 and October 25, 2019, and was organized in three steps (Figure 1). The choice of experimental sites was decided in agreement with the territorial community, with the main criterion of individual household districts.

In a first step (i.e., baseline, Time 1), recyclable waste from households (*N* = 197) was collected during a specific collection round. Each garbage can collected was identified by their household’s address. The waste was transferred to the characterization area. The characterization consisted of separating and measuring the sorting errors (i.e., food waste, used batteries, electrical devices, medicine, kitchen utensils, glass bottles, and so on) from correctly recycled waste (i.e., cardboard and paperboard packaging, plastic bottles, cans, and so on). These errors as well as the correct items were weighed separately before being returned to the regular waste-processing process. A core characterization team performed all the analyses in order to maintain consistency within the project. In a second step (i.e., intervention, Time 2), public authority officers were trained by the experimenter in one of the four interventions, each of them corresponding to one experimental condition. Public authority officers were then deployed in pairs and met the households collected in door-to-door interventions (Dai et al., 2015) during Time 1 by applying one of the four interventions (*N* = 139). In a third step (Time 3), a second waste collection and characterization were conducted 1 week after door-to-door interventions (post-measurement, *N* = 119). Collection and characterization steps were similar at Time 1 and Time 3.

#### Measurement (Time 1 and Time 3)

To evaluate the effectiveness of the interventions deployed in Time 2, we calculated the proportion of sorting errors by dividing the quantity of sorting errors (in kg) by the total weight of the waste (correct sorting and sorting errors, in kg).

##### Interventions (Time 2)

###### Material

**FIGURE 2:**
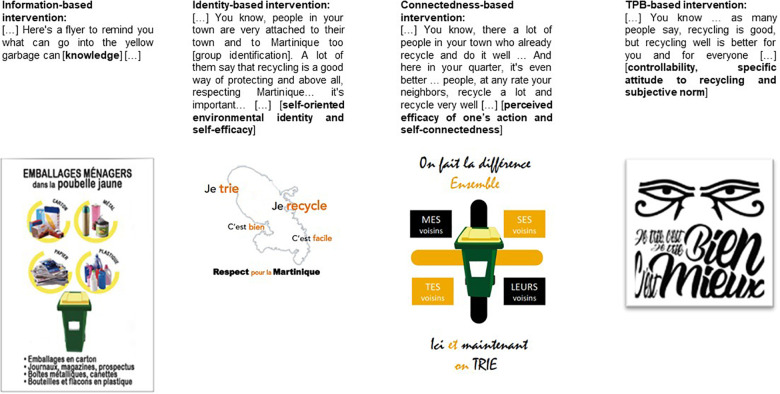
Stickers illustrating the combination of the determinants of recycling.

Based on the psycho-environmental diagnostic, four stickers were designed. Each of them illustrated the combination of determinants of intention (Figure 2). In addition to this, we also designed a communication script for the officers, in order to standardize the interventions.

###### Door-to-Door Intervention

Each communication script integrated binding communication elements, such as preparatory acts (Girandola, 2003; Girandola and Joule, 2012). Concretely, the public authority officers introduced themselves. The inhabitants answered a few questions and agreed to put out the recycling garbage can the following week (preparatory acts). These questions are important less for the information they provide than to encourage people’s commitment. Then, according to each intervention, public authority officers used one of the communication scripts (Figure 2). They distributed a sorting guide to the households reminding them of what waste was to be sorted. In three of the four interventions, the public authority officers suggested that the households put a sticker on their recycling garbage can.

### Results

#### Description of Households

In a survey, 197 cans of recycled waste were collected at Time 1. Following collection, the officers contacted 139 households door to door (Time 2). Out of these 139 households, 119 were collected at Time 3. The descriptive analysis of these 119 households (Table 5) shows that we do not observe any differences between the four conditions relative to age [*F*_(3,107)_ = 0.72, *ns*], number of people living in the household [*F*_(3,110)_ = 1.74, *ns*], number of households with children [Chi2 (3) = 0.41, *ns*], average number of children per household [*F*_(3,32)_ = 0.87, *ns*], and income [*F*_(3,105)_ = 2.63, *p* = 0.054, *ns*].

**TABLE 5:** Descriptive analysis of the sample among the four conditions of the 119 households.

	*N*	Age	Person per household	Number of households with children	Average number of children per household	Income per household
Information-based int.	33	54.9 (13.3)	2.83 (0.95)	15/33	1.53 (0.51)	4.9 (1.9)
Connectedness-based int.	30	59.8 (17.3)	2.54 (1.17)	6/30	1.67 (0.51)	4.85 (1.71)
Identity-based int.	31	60.3 (15.8)	2.36 (1.31)	8/31	1.63 (0.91)	3.77 (1.76)
TPB-based int.	25	59.1 (15.9)	3.04 (1.34)	7/25	2 (0.58)	4.58 (1.56)

*Standard deviations are reported in brackets.*

#### Results on the Quantities of Waste Produced

At time 1, 197 recycling garbage cans had been collected (583.65 kg). At T2, 119 recycling garbage cans had been collected (293.1 kg). In a survey, 60% of the garbage cans collected in T1 were collected and characterized in T3. This drop in sample size is observed in each experimental conditions (information-based intervention: 33/52, connectedness-based intervention: 30/50, identity-based intervention: 31/53, and TPB-based intervention: 25/42).

#### Results on the Proportion of Sorting Errors

At Time 1, sorting errors represented 201.4 kg out of a total weight of collected waste of 583.65 kg, for a sample of 197 households. The proportion of sorting errors was therefore 0.34. No significant difference between the four conditions on the proportions of sorting errors was observed (0.32 for the information-based intervention, 0.31 for the connectedness-based intervention, 0.43 for the identity-based intervention, and 0.29 for the TPB-based intervention). To evaluate the effectiveness of each of our interventions, we consider the overall proportion of rejected recycling waste (0.34) such as the standard of comparison.

Overall, the 119 households collected at time T3, sorting errors represented 79.75 kg out of a total weight of collected recyclable waste of 293.1 kg (0.27). All interventions combined, no significant difference in the proportion of sorting errors before and after the officers’ intervention was observed (*z* = 1.35, *p* = 0.09).

We compared the proportion of sorting errors at T1 (i.e., 0.34) with the proportion of sorting errors at T3, in each of the four interventions (Table 6). The results suggest that the first three interventions based on information, connectedness, and identity do not enable a significant reduction of sorting errors (respectively, *z* = 0.98, *p* = 0.16, *z* = 0.78, *p* = 0.21 and z = −0.39, *p* = 0.34). Solely the TPB-based intervention is efficient for decreasing sorting errors (*z* = 1.58, *p* < 0.05).

**TABLE 6:** Proportion of sorting errors among the four conditions at T2.

Intervention	*N*	Proportion of rejected recycling waste	Comparison to 0.34 (*N* = 197)
Information-based int.	33	0.25	*z* = 0.98, *p* = 0.16
Connectedness-based int.	30	0.27	*z* = 0.78, *p* = 0.21
Identity-based int.	31	0.38	*z* = −0.39, *p* = 0.34
TPB-based int.	25	0.18	*z* = 1.58, *p* < 0.05

## General Discussion

Our aim was to design and test evidence-based intervention to improve recycling behavior in Martinique. Two stages were necessary to reach this objective: carry out a psycho-environmental diagnostic to identify the determinants of recycling and implement them into a behavioral change intervention adapted to the island context. According to Varotto and Spagnolli (2017, p. 176) *“despite the fact that it might be considered time-consuming and/or expensive in the economics of a field intervention, it is instead important to connect the planning of the intervention with a deeper knowledge of the recipients and their characteristics, especially when the effectiveness of the intervention is based on the personalization of contents (e.g., information, feedback, recommendations, etc.).”*

First, the psycho-environmental diagnostic identified determinants of recycling already identified in the literature. In particular, the role of self-efficacy is a strong one (Geiger et al., 2019) and proves to be a good predictor of the intention to recycle, whatever the behavioral temporal dimension of change. Our results enable us to identify different determinants according to the temporality. As mentioned above, short-term recycling intention is based on concrete recycling aspects (i.e., knowledge) and self-oriented determinants (i.e., controllability and identity). On the other hand, medium-term recycling intention is determined by the abstract aspects of desirability (i.e., attitude) and social-oriented determinants (i.e., norm). These results are consistent with Trope and Liberman (2003, 2010): a proximal (vs. distal) temporal distance is associated with a proximal social distance (self-oriented) vs. distal (social-oriented).

Second, based on this psycho-environmental diagnostic, field research was conducted to test and compare the effectiveness of each of the four interventions. For this, we set up effective behavior change measurements (i.e., improvement of recycling quality). The intervention based solely on information (information-based intervention) did not prove to be very effective. Accordingly with Geiger et al. (2018), if informing is doubtless necessary to develop knowledge, it appears that it is not sufficient to trigger a behavior change. Subsequently, the interventions designed to make the identity or connectedness salient (respectively, identity-based intervention and connectedness-based intervention) did not prove to be very effective. Martinique is an island with strong multiculturalism, on which the salience of the Martinican identity may not be very relevant. Our results show that solely the intervention that articulates attitude, controllability, and subjective norm (TPB-based intervention) is efficient in improving recycling quality. Taken together, these dimensions made salient in the flyer and the communication script reflect the three determinants of intention and behavior, according to the TPB theory, taking the temporal dimension into consideration at the same time. Thus, our intervention focusing on the positive attitude toward recycling, its socially desirable dimension, and controllability is effective in influencing not only the intention but also the effective behavior. Once again, theory of planned behavior offers a relevant setting for conducting behavior change interventions (Steinmetz et al., 2016).

### A Framework for Application: Psychosocial Engineering Approach

In reference to the Stokes model (1997), our research-action takes place in the Pasteurian quadrant (Reich, 2008): socially useful and high scientific added value. It means designing applied research and fundamental research by considering them in a dynamic relationship. Instead of opposing them, psychosocial engineering assumes to articulate them closely, each one being both indispensable and at the service of the other, in order to respond to a societal demand. More precisely, psychosocial engineering is part of the idea that social psychology has the means to produce knowledge that gives social psychologists “engineering utilities” (Beauvois et al., 1989). Three arguments support this point of view. First, the approaches deployed in the field have a solid theoretical and methodological background built up through experimentation. Second, they require a close and complex analysis of the social context of the behavior to be changed. Third, they require the ability to design and implement concrete proposals for modifying the situation, and to understand the consequences of this modification.

Psychosocial engineering is based on the articulation of five iterative steps: (1) assessing behavior in the social context, (2) proposing alternatives for what already exists, (3) comparing them, (4) disseminating the selected alternative, and (5) accompanying field actors through professional training. Our intervention takes up the five steps. First of all, the psycho-environmental diagnostic enables the identification of the specificities of the island context, the motivational factors of recycling in this context, and the specification of the level of intervention, i.e., sorting in households (step 1). Based on this diagnostic, we formulated four theoretically founded proposals (step 2) before comparing them in the field (step 3). The most effective intervention (TPB-based intervention) is our action model (step 4). The challenge of step 5 is to facilitate the appropriation of the action model by the officers. This appropriation is operated by the design and animation of training for these actors. The articulation of these iterative steps between the officers’ professional practices and the development of scientific knowledge ended in the restitution of the results to all the partners involved (public policies, collection service partners). As Norström et al. (2020) concluded, research aimed at addressing sustainability challenges is most effective when “co-produced” by academics and non-academics.

### Limits and Perspectives

Several limits inherent to this research can be mentioned. First, the psycho-environmental diagnostic was based on an online questionnaire on the local authorities’ website. However, only those who already showed an interest in this issue answered (i.e., sample bias). Moreover, the measurement of recycling intention should be considered as weak as Ma et al. (2021) have pointed out. In addition to this, TPB model allows the inclusion of additional variables to improve the predictive power of the theory (Yuriev et al., 2020). The flexibility of the model made it possible to introduce variables specific to the island context (e.g., sense of community) and identities. A growing body of research suggests a link between religiousness and environmental attitudes or behaviors (Arbuckle and Konisky, 2015; Arli et al., 2021). In the island context of our intervention, religiousness has an important place in the daily lives of Martinicans. Future research will integrate this dimension. Lastly, future research would benefit from enlarging the role of the temporal distance on the intention to change behavior and integrating other dimensions of psychological distance such as social distance. The specificity of waste recycling lies in the fact that recycling is a private behavior that enters the public sphere as soon as people leave their garbage can on the sidewalk.

Secondly, regarding evidence-based intervention, we have faced challenges related to the field. Observing the behavior of households shows that they put out the recycling garbage can when it is full, and not every week. However, in this research action, the households were asked to put out their recycling garbage can at the time of the door-to-door intervention (i.e., between two collections). In other words, if we are sure that the collection and the characterization in week 2 (post-intervention) correspond to 1 week of waste production, we are less certain regarding the weekly collection in week 1 (pre-intervention), which may contain more than 2 weeks of recycling. Lastly, the long-term effects of change are an important issue. The psychosocial engineering approach aims to maintain and reinforce the appropriation of new professional practices (here the officers’ practices) through professional training. Regular intervention should also be repeated with households to maintain recycling at the expected level.

## Data Availability Statement

The raw data supporting the conclusions of this article will be made available by the authors, without undue reservation.

## Ethics Statement

Ethical review and approval were not required for the study on human participants in accordance with the local legislation and institutional requirements. The patients/participants provided their written informed consent to participate in this study.

## Author Contributions

Both authors wrote the initial draft, equally contributed to subsections of the manuscript, led the conceptual design of the manuscript, reviewed the manuscript, provided comments and feedback, and approved the submitted version.

## Conflict of Interest

The authors declare that the research was conducted in the absence of any commercial or financial relationships that could be construed as a potential conflict of interest.

## Publisher’s Note

All claims expressed in this article are solely those of the authors and do not necessarily represent those of their affiliated organizations, or those of the publisher, the editors and the reviewers. Any product that may be evaluated in this article, or claim that may be made by its manufacturer, is not guaranteed or endorsed by the publisher.

## Footnotes

1 The sample was composed of the 18 municipalities of the territorial authority, with a good proportional representation of the population of each municipality.

2 According to Field’s recommendations (Field, 2009), if you want to test the model overall, you need 50 + (8 × number of predictors), i.e., 50 + (8 × 12) = 146. Most of the time, you also need to test the individual predictors. Then, Green [1991, cited by Field (2009)] suggested a minimum sample size of 104 + number of predictors, i.e., 104 + 12 = 116. Moreover, for 20 predictors or fewer, a sample size of 200 will always suffice, if a medium-size effect was expected (Field, 2009). Our sample of 213 respondents is therefore sufficient.
